# A Molecular Approach to Identifying the Natural Prey of the African Creeping Water Bug *Naucoris*, A Potential Reservoir of *Mycobacterium ulcerans*

**DOI:** 10.1673/031.012.0201

**Published:** 2012-01-09

**Authors:** Maribet Gamboa, Ryan K. Kimbirauskas, Richard W. Merritt, Michael T. Monaghan

**Affiliations:** ^1^ Leibniz - Institute of Freshwater Ecology and Inland Fisheries (IGB), Müggelseedamm 301, 12587, Berlin, Germany; ^2^ Department of Entomology, Michigan State University, East Lansing, Michigan, 48824, USA

**Keywords:** Buruli ulcer, cloning, DNA barcoding, food web, Naucoridae, prey

## Abstract

The extra-oral digestion of creeping water bugs (Naucoridae: Hemiptera) hinders the study of their diet using the standard method of identifying prey body parts in the gut. Genetic methods are available, but rely on PCR tests or similar diagnostics to confirm suspected prey. Where the potential prey is unknown and a broad search for all possible prey is desirable, methods that can potentially capture any prey item are required. *Naucoris* sp. is known to harbor *Mycobacterium ulcerans* (Actinomycetales: Mycobacteriaceae), the causative bacterium of Buruli ulcer. Outbreaks of Buruli ulcer have been associated with disturbed freshwater habitats, but the mode of transmission to humans remains unclear. Here we examine the diet of *Naucoris* sp., a dominant aquatic predator in water bodies in Ghana where the prevalence of Buruli ulcer is high. We cloned and sequenced 576 PCR products (mtDNA rrnL, cox1) isolated from the gut of 60 *Naucoris* sp. individuals to determining diet composition as broadly as possible. Using phylogenetic analysis of newly sequenced clones and 6 potential prey taxa collected from the site, sequences isolated from *Naucoris* sp. guts matched locally collected Coleoptera (Hydrophilidae). Blastn queries to GenBank of other clone sequences produced matches to (Anura) (n = 1), Rotifera (n = 5), and fungi (n = 4) as additional components of the diet. Our results suggest that sp. in this Buruli ulcer-endemic area feeds on a wide range of prey and body sizes, and that the approach could be successfully applied to studies of aquatic food webs where morphological identification of prey is impossible and where little or no a priori knowledge is available.

## Introduction

Most Hemiptera feed by injecting digestive enzymes into prey and then ingesting the liquefied tissues through a tube-like proboscis (extra-oral digestion) (Cohen 1995). This feeding mode presents a challenge to the study of their diet, largely eliminating the use of standard morphological identification of chitinous body parts in the gut. As a result, studies of hemipteran diets have typically used immunoassays employing prey-specific monoclonal antibodies (Greenstone 1996), PCR tests (Sheppard and Harwood 2005), or both (e.g., Fournier et al. 2008). One limitation of most antibody- and DNA-based applications is that some knowledge of the potential prey is required. Antibodies target epitopes that are specific to proteins from target prey species, and most DNA methods employ species- or taxon-specific primers in PCR tests to determine the presence/absence of target prey species or taxa (e.g., Agustí et al. 1999, 2000, 2003; Read 2002; Cuthbertson et al. 2003; Jarman et al. 2004; de León et al. 2006). While these methods can be powerful and have been verified for their accuracy using laboratory feeding experiments (Chen et al. 2000; Foltan et al. 2005; Harper et al. 2005, 2006; Sheppard et al. 2005; Harwood et al. 2007; McMillan et al. 2007), a major limitation arises when there is little or no prior knowledge of the prey in their natural habitat.

Buruli ulcer is a neglected emerging disease of skin and soft tissue that leads to scarring and disability (Johnson et al. 2005; Merritt et al. 2005). It is caused by *Mycobacterium ulcerans* (Actinomycetales: Mycobacteriaceae), an environmental pathogen that produces a destructive polyketide toxin (George et al. 1999). The disease has been reported in humans from at least 32 countries, with a large number of cases reported from West Africa (Duker et al. 2006; Walsh et al. 2008; Merritt et al. 2010). While transmission of the disease to human beings remains unclear, Buruli ulcer outbreaks have been associated with freshwater habitats (Thangaraj et al. 1999), particularly in areas where the landscape is disturbed by natural events such as flooding, or through deforestation, dam construction, agricultural diversion, or mining (Thangaraj et al. 1999; Johnson et al. 2005; Merritt et al. 2005; Duker et al. 2006; Wansbrough-Jones and Philips 2006).

A critical step in understanding *M. ulcerans* transmission is elucidating the diet of organisms that may potentially act as reservoirs and vectors of the pathogen in nature. Non-human mammals and reptiles have been tested in the environment without positive findings for the pathogen (Radford 1974), and several arthropod disease vectors (i.e., bedbugs, black flies, mosquitoes) tested negative in early studies (Revill and Barker 1972; Portaels et al. 2001). However, only a few organisms in each taxonomic group were tested in these early studies, and insect sampling methods were neither systematically employed nor standardized. Portaels et al. (1999) were the first to suggest that aquatic bugs (Hemiptera) might be reservoirs of *M. ulcerans* in nature, and recently they described the first isolation in pure culture of *M. ulcerans* from a water strider (Hemiptera: Gerridae, *Gerris* sp.) from Benin, West Africa (Portaels et al. 2008). A survey study (Portaels et al. 2001) based on detecting *M. ulcerans* DNA in aquatic insects (Hemiptera, Odonata, Coleoptera) in African Buruli ulcer-endemic swamps confirmed the earlier findings. More recent studies in Australia have suggested that mosquitoes may be involved in transmission (Johnson et al. 2007).

**Figure 1.**
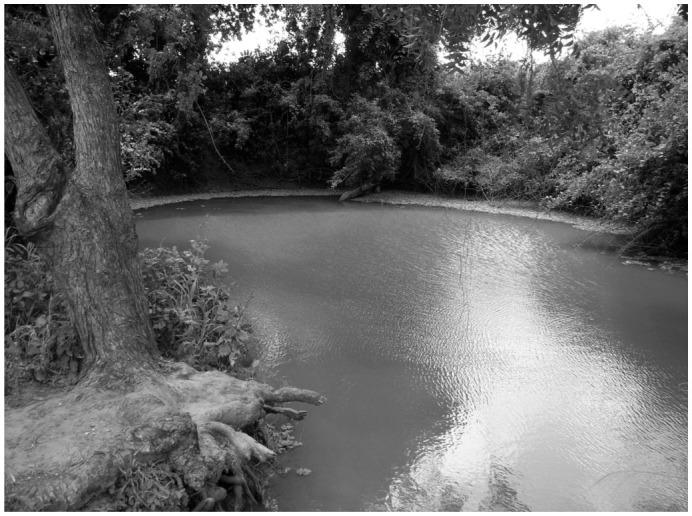
Photograph of the study site near the village of Saduase, Ga East District, Ghana. High quality figures are available online.

Using experiments, it has been demonstrated that *M. ulcerans* could survive and multiply within the salivary glands of the aquatic bug *Naucoris cimicoides* (Hemiptera, Naucoridae), and that *N. cimicoides* could transmit the mycobacteria to mice (Marsollier et al. 2002, 2003). *Naucoris* spp. (Naucoridae, Hemiptera) live in freshwater ponds, lakes, and slow-flowing sections of streams and rivers. Naucoridae are predacious in both the immature (nymph) and adult stages, although little is known of the ecology and prey preferences of Naucoridae in nature. This is particularly true in tropical West Africa where the disease is prevalent. Most aquatic hemipterans are believed to be generalist predators on other aquatic invertebrates (Merritt et al. 2008), although some, including naucorid species, have mouthparts designed to aid in feeding on prey larger then themselves (e.g., Cohen 1995) such as tadpoles (Polhemus and Polhemus 1988) and larval fish (Louarn and Cloarec 1997).

Here we examine the diet of an abundant predator in Buruli ulcer-affected freshwater ecosystems in West Africa (*Naucoris* sp.) in a first attempt to understand its role in a tropical pond food web and potential sources of *M. ulcerans.* In the absence of any *a priori* knowledge of their prey, we used PCR to amplify all DNA in the *Naucoris* sp. gut using universal primers, clone the PCR product, and sequence a subset of clones. We then matched the resulting gut-content sequences to sequences obtained from potential prey collected from the same habitat, and to publically available sequence databases.

## Materials and Methods

### Sample collection and preparation

*Naucoris* sp. water bugs and potential prey populations were sampled 9 August 2009 from one body of water within the village of Saduase, Ga East District, Ghana (Figure 1). All macroinvertebrates were collected using a 500 µm D-frame net. All *Naucoris* sp. were transferred immediately to individual vials, while all other macroinvertebrates were considered to be potential prey items and stored separately. All specimens were preserved in 95% ethanol in the field. In the laboratory, *Naucoris* sp. were sexed and guts were carefully removed under a dissecting microscope. To expose the guts, heads were dissected and incisions were made laterally along the abdomen to peel back the exoseleton. Guts were then removed with forceps and stored separately in fresh 95% ethanol. Prior to handling each specimen, all instruments were rinsed with distilled water, flame treated, and wiped with individual Kimwipes. All samples were stored at 4°C prior to DNA extraction.

### DNA extraction and PCR

Genomic DNA was extracted from *Naucoris* sp. adults (*n* = 29) and nymphs (*n* = 14) as well as the potential prey sampled: Ephemeroptera (mayflies, *n* = 4), Odonata (damselflies, Zygoptera) (*n* = 8), Coleoptera (beetles, *n* = 4), Diptera (flies, Chironomidae, *n* = 3), and Arachnidae (spiders, *n* = 3) using DNeasy tissue kits (Qiagen GmbH, Hilden, Germany). Genomic DNA was extracted from *Naucoris* sp. guts (*n* = 60) using the QIAamp DNA Stool Mini Kit (Qiagen GmbH, www.qiagen.com) (King *et al.* 2008). Field samples were first centrifuged for one min at 2000 g. The ethanol was poured off and the dry weight of the pellet was determined. All remaining steps followed the manufacturer's protocol, except that only half the recommended volume of buffers/InhibitEX was used. Primers LR-N-13389 (alias 16ar, Simon et al. 1994) and 16b2 (5′TTTAATCCAACATCGAGG-3′) were used to amplify a ca. 440-bp fragment of mitochondrial *rrnL* (16S) for all samples using standard methods. The 5′ (DNA barcode) region of *coxl* (COI) was amplified for four *Naucoris* sp. adults using primers LCO-1490 and HCO-2198 (Folmer et al. 2004) in order to potentially match individuals with existing databases. PCR products were purified using the QIAquick PCR Purification Kit (Qiagen GmbH, www.qiagen.com) and sequenced in both directions using the PCR primers. Samples were analyzed on either a CEQ 8000 (Beckman/Coulter, www.beckmancoulter.com) or a 3500xL (Applied Biosystems, www.appliedbiosystems.com) automated sequencer.

### Molecular cloning

We used cloning to differentiate among multiple possible PCR products obtained from *Naucoris* sp. guts. Both the *rrnL* and *coxl* PCR primers (above) target a broad range of organisms including crustaceans, insects, and vertebrates, thus could be useful for simultaneously amplifying multiple taxa that may be present in the gut. PCR products were run out on a 2 % agarose gel and purified using the MinElute gel extraction kit (Qiagen GmbH, www.qiagen.com). The clone libraries were created with the pGEM-T-Easy-kit (Promega GmbH, www.promega.com) following the manufacturer's protocol. Insert size was examined using PCR of plasmids with the primers SP6 (5′-ATTTAGGTACACTATAG-3′) and T7 (5′AATACGACTCACTATAGG-3′). Large inserts (*n* = 576) were cleaned with PEG and sequenced using primer SP6.

### Data analysis

All sequences were assembled and edited using CodonCode Aligner v 3.5 (Codon Code Corporation, www.codoncode.com). For *Naucoris* sp. and prey, forward and reverse sequences were assembled and edited for each specimen. *Naucoris* sp. *coxl* and *rrnL* sequences were first compared to the NCBI nucleotide database using blastn queries (http://blast.ncbi.nlm.nih.gov). Clone sequences were assembled and contigs were edited in order to generate consensus sequences for each contig (contig sizes see Table 1). All full-length sequences obtained from clone libraries (*ca.* 450–478 bp *rrnL*, 658 bp *coxl*) were compared to the NCBI nucleotide database using blastn queries.

Phylogenetic analysis was conducted on all insect and arachnid *rrnL* sequences that were newly generated by direct sequencing or cloning. We also included 18 other Hemiptera *rrnL* sequences obtained from the list of blastn hits, most of which came from a recent mtDNA phylogeny (Hua et al. 2009). For comparison of our potential prey sequences with the NCBI database, we downloaded all blastn hits with > 90 % identity to each query genotype. All sequences were aligned using clustalW (align.genome.jp) and a phylogenetic tree search was conducted on the matrix using a maximum likelihood approach in PhyML v 3.0 (Guindon and Gascuel 2003) under a GTR model of evolution (as determined by Modeltest v 3.7, Posada and Crandall 1998).

## Results

**Figure 2.**
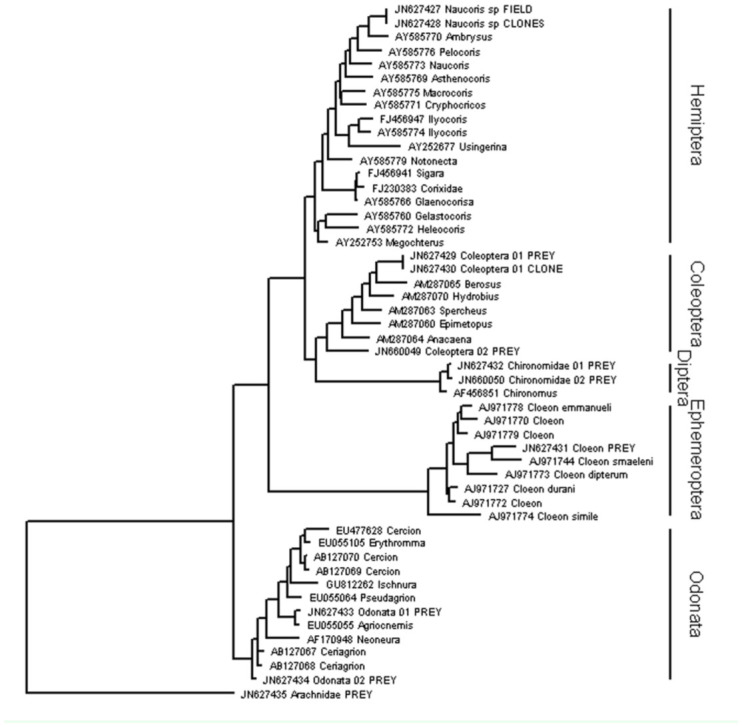
Maximum likelihood *rrnL* gene tree reconstructed using a GTR model of evolution, including newly sequenced *Naucoris* sp. (terminals labeled FIELD), potential prey (PREY), cloned PCR products from *Naucoris* sp. guts / mouthparts (CLONES), and highly ranked sequences according to blastn queries (with GenBank alphanumeric accession codes, see text for criteria). High quality figures are available online.

PCR amplification of *coxl* and *rrnL* was equally successful for *Naucoris* sp. but *rrnL* was more consistently amplified for gut samples and potential prey. There were no *Naucoris* sp. *coxl* sequences available on the NCBI nucleotide database and the top hit of the blastn query was an unclassified Hemiptera (AAG5301 voucher ENT-OUBS-156, HM381306), whereas the database contained 8 *rrnL* sequences for Naucoridae collected from Madagascar, Europe, North and Central America, and the Philippines (Hebsgaard *et al.* 2004). Combining the newly generated *rrnL* sequences with blastn query results produced an aligned matrix of 62 taxa and 460 characters (sequence length 336–443 bp). In the maximum likelihood *rrnL* gene tree (In L = -8123.81514 ; Figure 2) our *Naucoris* sp. sequences were clearly nested within published data for the Hemiptera, the closest relative being *Ambrysus* sp. collected from North America (Figure 2). *Ambrysus* sp. was the second-ranked blastn hit, with *Macrocoris* sp. from Madagascar (Hebsgaard *et al.* 2004) the top hit but phylogenetically more distant in our analysis of the same sequences (Figure 2).

**Table 1.**
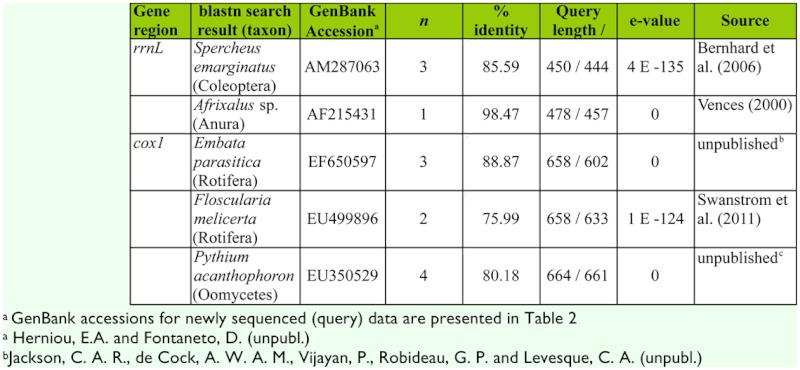
Results of comparisons of *rrnL* and *coxl* sequences that were PCR-amplified from *Naucoris* sp. guts and cloned (see Methods) to the NCBI nucleotide database using blastn queries. Taxa listed were, in each case, first on the hit table. Only full-length inserts (450 – 478 bp rrnL, 658 bp coxl) were used as query sequences. Other full-length inserts were identical to our sequences obtained from direct sequencing of sampled Naucoridae and resulted in a top blastn hit of *Macrocoris* sp. (*rrnL*) or Hemiptera sp. (*coxl*) (data not shown).

**Table 2.**
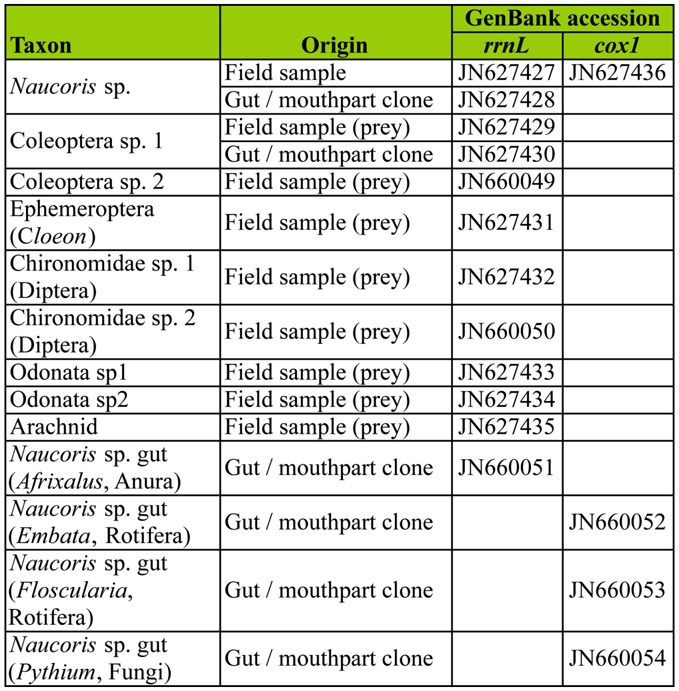
Accession numbers of newly sequenced material including taxa (with closest match using blastn searches in parentheses) and sequence origin (directly sequenced organism, or sequenced clone from gut/mouthpart content).

Three of our cloned *rrnL* sequences from Naucoris sp. guts were identical to a field-caught Coleoptera species sampled at the study site as potential prey. The top database match (using blastn) to this sequence was *Spercheus* (Spercheidae) (Table 1) and the second-ranked match was *Hydrobius* sp. (Hydrophilidae) (not shown), but it is clear from our phylogenetic search that it is distantly related to both based on mtDNA *rrnL* (Figure 2). None of the other potential prey species that we collected from the sampling site and sequenced were recovered from gut sequence clones (Figure 2). Nonetheless, a number of interesting non-insect species were recovered from guts and identified with blastn queries (Table 1). These included *Afrixalus* sp. (Anura: Hyperoliidae), a sub-Saharan genus of frog for which we recovered one *rrnL* sequence from our clone library. Sequences from the *coxl* clone library included *Embata* and *Floscularia* (Rotifera), and *Pythium* (Oomycete fungi). All other fulllength clone sequences were identical to our Naucoridae sequences obtained using direct sequencing of PCR products (*rrnL* shown in Figure 2).

## Discussion

Buruli ulcer (*Mycobacterium ulcerans* infection) causes severe morbidity in human populations associated with degraded freshwater habitats, but neither the reservoir nor the mode of transmission of *M. ulcerans* is known (Merritt *et al.* 2005, 2010). Here we investigated an abundant aquatic predator from a Buruli ulcer-endemic area in Ghana, *Naucoris* sp. water bugs (Hemiptera, Naucoridae). While Naucorids have been implicated in the transmission of *M. ulcerans* in laboratory studies, a limited knowledge of their place in aquatic food webs in nature makes it difficult assess the potential source and sinks of the pathogen. This is the first investigation of which we are aware to clone and sequence PCR products from universal primers to determine a Hemipteran diet without any prior knowledge of potential prey.

Our approach led to a broader perspective of the role of *Naucoris* sp. in the aquatic food web. Using a standard PCR-based method, we would have designed prey-specific primers for the 5 taxa of field-caught prey (e.g., Agustí et al. 2003). From this, we would have probably generated positive tests for the Coleoptera. In contrast, the universal primers, sequenced clones, and database queries used here allowed us to identify DNA in the guts that came from taxa that were not field-collected. These included fungi, rotifers, and an anuran, although larval anurans were collected from the field site and thus known to be present. A limitation of the database queries is clearly the fact that the extent of the database plays an important role. Using the newly generated *Naucoris* sp. sequences, none of our blastn query results gave a close match. Even our “barcode” *coxl* sequence gave a fairly meaningless match (Hemiptera sp.) to the Barcode of Life database (www.barcodinglife.org).

Combining public databases and our own newly generated prey sequences was beneficial for confirming that the prey Coleoptera sequence cloned from the gut matched the field-caught Coleoptera species at the same habitat. Although neither the database nor our new sequence could provide identification, at least we could conclude with some confidence that the *Naucoris* sp. preys on the resident Coleoptera. These sequences were identical and, based on the phylogenetic gene tree, quite different from any species in GenBank. Interestingly, the blastn query and phylogenetic analysis gave different results. The second-ranked blastn result was phylogenetically closer to the query sequence than the top-ranked blastn query result. The more detailed phylogenetic analysis, using more of the available data and a GTR model of sequence evolution, likely revealed the closer relative. Although both query hits were relatively distant and neither is probably a good match, it does suggest that a phylogenetic approach is more accurate than a blast result in the absence of a complete database.

In conclusion, our approach provided the means to study an aquatic hemipteran diet without any prior knowledge of potential prey and despite the difficulties of extra-oral digestion. *Naucoris* sp. in this Buruli ulcerendemic area feeds on a wide range of prey and body sizes, including rotifers, insects, and anurans. Further work on *M. ulcerans* transmission will be aided by this food web information. Our results also suggest the approach could be successfully used to study the complex interactions within aquatic food webs, including even feeding on fungi. Our results corroborate previous suggestions that DNA-based approaches using universal primers and cloning provide an important tool for studying the prey spectrum of predators with unknown diets.
